# Knowledge, attitude, and practices to zoonotic disease risks from livestock birth products among smallholder communities in Ethiopia

**DOI:** 10.1016/j.onehlt.2021.100223

**Published:** 2021-01-30

**Authors:** Gezahegn Alemayehu, Gezahegne Mamo, Hiwot Desta, Biruk Alemu, Barbara Wieland

**Affiliations:** a International Livestock Research Institute (ILRI), Addis Ababa, Ethiopia; b Department of Microbiology, Immunology and Veterinary Public Health, College of Veterinary Medicine and Agriculture, Addis Ababa University, Bishoftu, Ethiopia; c College of Veterinary Medicine, Samara University, Samara, Ethiopia

**Keywords:** Livestock birth products, Differential item functioning, Ethiopia, KAPs, IRT, Zoonotic disease

## Abstract

Many causes of abortion in livestock are due to zoonotic pathogens that pose serious infection risks for humans. Carefully designed, empirical One Health research allows to untangle the complexity around these risks and guides the development of practical health education guidelines and best prevention practices for veterinary public health interventions. To support this, the study presented here aimed at understanding knowledge, attitudes, and practices (KAP) on zoonotic risks from livestock birth products among rural communities in Ethiopia. From July 2018 to February 2019, a cross-sectional study design was conducted with 327 randomly selected farmers and pastoralists in five districts in three regions in Ethiopia. The structured questionnaire consisted of 48 items to evaluate knowledge (24), attitude (9), and prevention practices (15) related to zoonotic diseases risks from livestock birth products. A unidimensional two-parameter logistic (2-PL) Item Response Theory (IRT) model was used for zoonotic disease risk KAP scale construction and evaluation. The 2-PL IRT model was fitted to determine the probability of a person to appropriately respond to an item with a provided zoonotic disease KAP level. We then examined differential item functioning (DIF) concerning to five important covariates. The attitude subscale had the highest total mean score (37.3, ± 28.92%) and the knowledge subscale had the lowest mean score (22.4, ± 33.6%) among the three subscales. The mixed model regression analysis indicated that region was the only apparent factor explaining differences in zoonotic diseases knowledge, attitude, and practice total mean scores. The knowledge and attitude subscales had good internal consistency with a Cronbach's α at 0.83 and 0.81, respectively, whereas the practice subscale had lower internal consistency with 0.51. There was a positive association between responding to knowledge questions correctly and a positive attitude (r2 = 0.44, *p* < 0.0001) and self-reported good practice (r^2^ = 0.307, p < 0.0001). The differential item functioning test showed that 19 of 37 (51.35%) and 12 of 37 (32.43%) items of the retained KAPs survey items had non-uniform and uniform DIF linked to at least one covariate respectively and all the covariates were related with DIF in at least one item. This study found substantial knowledge gaps, a low level of the desired attitude, and high-risk behavioural practices regarding zoonotic disease from livestock birth products. Consequently, livestock keepers are likely exposed to pathogens and thus these practices are an important contributing factor for zoonotic disease infection in people.

## Highlights

• Use 48 items to evaluate zoonotic diseases risks KAP from livestock birth products.
• Item Response theory was used for KAP scale construction and evaluation.
• Respondents' knowledge positively associated with desired attitude and practices.
• Low zoonotic diseases knowledge, desired attitude and higher risky practices.
• 19 of 37 and 12 of 37 items had nonuniform and uniform DIF respectively.

## 1 Introduction

Livestock are important for the livelihoods of rural households in Ethiopia and contributes to food security and improves the income and wellbeing of farmers and pastoralist families [50,61]. Nevertheless, keeping livestock can also be a source of infection for humans [34]. In rural parts of Ethiopia, almost all household members have daily direct contact with animals and are involved in different stages of the animal production cycle [33]. Therefore, they likely face daily exposure to zoonotic pathogens. Close interaction between livestock and humans also occurs due to the close proximity of livestock to living accommodation or even shared housing during severe weather conditions [59]. Furthermore, limited public and livestock health services in the areas and poor zoonotic diseases prevention practices increased the risk of households' exposure to those agents and pose a significant public health threat to the farming community [37].

Abortion in livestock can be caused by zoonotic pathogens and affects the individual animal and overall herd productivity [42]. In addition to a significant economic impact on the rural economy, many of the infectious agents that cause animal abortion have the potential to cause serious illnesses in people -in particular *Toxoplasma gondii*, *Brucella spp*, *Chlamydophila spp.*, *Campylobacter spp.*, *Salmonella spp*, *Listeria spp.*, *Leptospira* spp. and *Coxiella burnetii* (the cause of Q-fever) [18,23]. Animal to human transmission mainly results from direct contact with infected materials such as uterine discharge, aborted foetus, and placenta infected while assisting with animal delivery and while caring for the new-born, or through dealing with abortion cases or exposure to a contaminated environment with abortion products [28,40]. Animal birth products from an infected dam carry a large number of these pathogens, which can be a source of infection for an entire household because of their close contact with livestock in unsanitary conditions [13,22].

There is increasing evidence that zoonotic causes of animal abortion are prevalent and widely spread in all livestock production systems in Ethiopia [17,19,20,25,54,56,57]. However, prevention and control of these zoonotic diseases remain largely neglected in most African countries. This is largely related to a poor understanding of the problem, the non-existence of appropriate diagnostic facilities and trained human power as well as lack of suitable and long-lasting zoonotic diseases prevention and control strategies [21,59].

The absence of rigorous zoonotic disease prevention and control programs poses a high risk to vulnerable poor rural livestock producers and livestock products consumers along the value chain. Lack of awareness, combined with poverty, means that risky behaviours related to animal management and abortion material handling and disposal persist [21]. Lack of knowledge implies the need for carefully designed, empirical research that considers the animal, environment, and human health domains in a One Health approach to untangle the complexity and enhance the development of practical health education guidelines and best prevention practices for veterinary public health intervention designs.

A zoonotic diseases control plan largely depends on public health awareness which can help in lowering the risk of infections along the livestock production and processing chain. Changing behavioural practices of high-risk groups through better public health education is critical to contain the spread of zoonotic infection from animal to the human population in resource-scarce settings [26,60]. The evaluation and description of KAP among farmers and pastoralists concerning zoonotic disease risk from the livestock birth products can help in designing and implementing effective zoonotic disease prevention and control strategies in the livestock population and in developing and executing more efficient community-based health education for livestock producers. Therefore, this study aimed at understanding knowledge, attitudes, and practices (KAP) on zoonotic risks from livestock birth products among three rural communities in Ethiopia.

## 2 Material and methods

### 2.1 Study design and setting

**Fig. 1:**
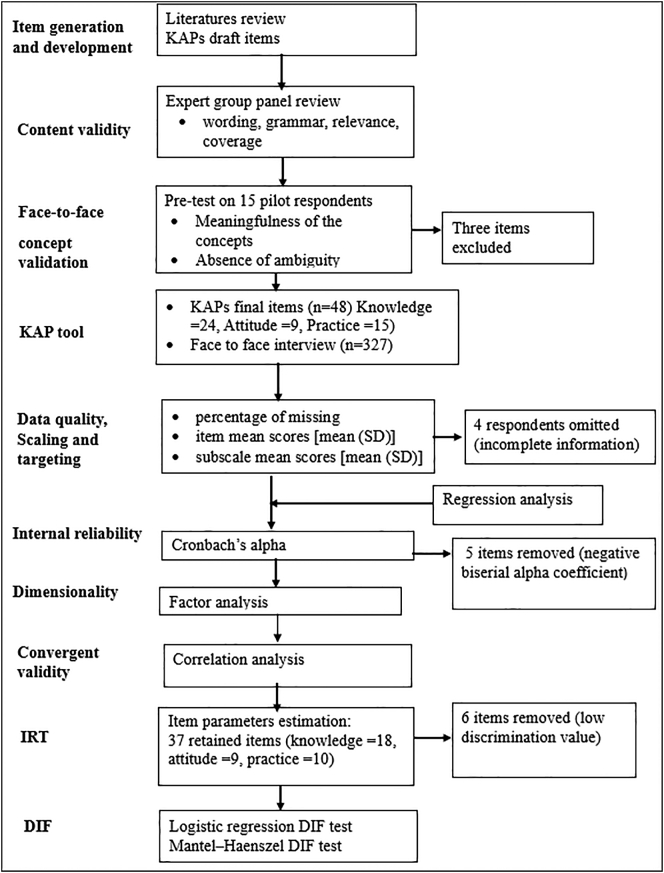
Study flowchart.

A cross-sectional study design was used to collect information from July 2018 to February 2019 in five locations (districts) in three regional states in Ethiopia, namely, Amhara, Oromia, and Southern Nation, Nationality and People (SNNP)(Fig. 1). The study sites were purposively selected to represent different agroecological conditions and production systems. Sites in SNNP region represented the highland agroecology, whereas sites in Oromia and Amhara regions represented the lowland agroecology. Mixed crop-livestock production is the predominant economic activity in Ancha district in SNNP, and in Abergelle and Zequwala districts in Amhara region. Livestock keeping as the predominant economic activity was the case in Yabello and Eleweya districts in Oromia region. In each of the five districts, two *kebeles* (= smallest administrative unit in Ethiopia) were selected, of these one kebele was a site included in the research of the CGIAR research program (CRP) on Livestock and had seen previous livestock value chain interventions, and one kebele had not previously seen any livestock interventions. The CRP Livestock intervention *kebeles* had received animal health intervention such as vaccination and community based internal parasite control program and training on herd health management (control of respiratory and reproductive diseases).

### 2.2 Participants

A total of 327 randomly selected participants were interviewed in the 10 targeted *kebeles.* The required sample size was calculated using an expected Cronbach's α of 0.7 with a significance level = 0.05; confidence interval of 95%; 24 response items (for knowledge sub-scale); and an expected dropout rate (incomplete information rate) of 10% [6,12]. The calculated sample size was equally distributed among the *kebeles*. Lists of potential participants in each *kebele* were obtained through local field researchers of CRP Livestock intervention *kebeles* and from key informants in the non-CRP Livestock kebeles one day before the survey. From these lists, households were selected randomly using the random function in Excel. Facilitators then contacted the household heads, asked for their willingness to participate, and planned the timing of the interview. Interviews were made in a place where both the interviewer and the participant felt comfortable and consent from all participants was obtained before the interview.

### 2.3 Survey instrument

A questionnaire was developed to measure participants' knowledge about zoonotic disease, attitudinal barriers related to zoonotic disease risk and exposure prevention from livestock birth products, and practices used to prevent zoonotic diseases risks from livestock birth products. The demographic questionnaire included information on gender, age, education, primary livelihood activity, ethnicity, marital status, intervention status, and residential area, as well as on animal abortion history in the flock.

The first author conducted a thorough review of the literature to generate items for the KAP survey. Then, to confirm the content validity of the survey tool, a panel of five experts (two veterinary public health specialists, two epidemiologists, and one animal production and health expert) evaluated the questionnaire in terms of wording, grammar, relevance, and coverage. The survey tool was tested on 15 farmers who were not included in the study population. The pilot study helped to assess the face validity of the items and to understand the meaningfulness of the concepts in the studied population. The difference and clarity of respondents' answers for each item were also evaluated. The survey tool was updated based on the feedback received during the pre-test.

The final version of the KAP tool comprised 48 items in three subscales (Table 1); 24 items measuring zoonotic diseases knowledge, 9 items measuring zoonotic disease risk attitude, and 15 items measuring zoonotic disease prevention practices. Of the 24 knowledge subscale items, 20 items had dichotomous responses (correct or incorrect) and 4 items were initially open-ended questions and then restructured into “correctly named” vs “not named correctly”. Items related to attitude were measured on a Likert scale ranging from 1 to 5 (1 = strongly disagree to 5 = strongly agree), with higher scores indicating the most desired attitude. All items in the practice subscale had dichotomous (“success” vs. “failure”). The questions were coded using Epi Info™ 7.2.1.0 software and copied onto Galaxy Table A (2016) for digital data collection.

**Table 1:** KAPs items description.

Item no	Item content	Response
Knowledge subscale items		
K1	When animals are sick in your flock, you can get the same sickness.	Correct/incorrect
K2	Many animal diseases can be transmitted from animals to humans	Correct/incorrect
K3	Identified the name of three diseases correctly that transmitted from animals to humans	Name /not name
K4	Identified the name of only two diseases correctly that transmitted from animals to humans	Name/ not name
K5	Identified the name of only one disease correctly that transmitted from animals to humans	Name/not name
K6	Please list at least one symptom for any one zoonotic disease in animals	Correct/incorrect
K7	Animal disease can be transmitted via different routes	Correct/incorrect
K8	Eating uncooked meat can transmit diseases from animals to you	Correct/incorrect
K9	Drinking of raw milk can transmit diseases from animals to you	Correct/incorrect
K10	Close contact with sick/dead animal can transmit diseases to you	Correct/incorrect
K11	You can get infection from environment contaminated from secretions of sick animals	Correct/incorrect
K12	Insect bite can transmit animal diseases to you	Correct/incorrect
K13	Animal bites can transmit diseases to you	Correct/incorrect
K14	Animal abortion can cause a serious economic and public health problem	Correct/incorrect
K15	Abortion in animals can be caused by agents that spread between animals	Correct/incorrect
K16	Infectious diseases that cause abortion in animals might cause abortion in humans	Correct/incorrect
K17	Abortion causing agents can pass to you through different routes	Correct/incorrect
K18	Name at least one abortion causing agents that is transmitted from animals to humans	Name /not name
K19	Assisting animals during parturition with bare hand exposes you to diseases	Correct/incorrect
K20	Assisting new-borns right after delivery exposes you to diseases	Correct/incorrect
K21	Any contact with aborted materials can expose you to diseases	Correct/incorrect
K22	Collecting aborted fetuses and placenta with bare hand exposes you to diseases	Correct/incorrect
K23	Disposing aborted fetuses into the environment can spread the diseases	Correct/incorrect
K24	Animal abortion can be prevented	Correct/incorrect

Attitude subscale items		
At1	Some animal diseases are dangerous for people	SDA/ DA/ N/ A/ SA
At2	Diseases that cause animal abortion are serious and need highest consideration	SDA/ DA/ N/ A/ SA
At3	Assisting the animal in delivery with bare hand can expose you to disease risks.	SDA/ DA/ N/ A/ SA
At4	Collecting the aborted fetuses and placenta with bare hands can expose you to disease risks	SDA/ DA/ N/ A/ SA
At5	Throwing aborted fetuses and placenta to the environment contribute the spread of the diseases in your farm	SDA/ DA/ N/ A/ SA
At6	You are at risk of acquiring diseases from abortion causing agents	SDA/ DA/ N/ A/ SA
At7	Many of the agents that cause abortion in animals have the potential to cause disease in people.	SDA/ DA/ N/ A/ SA
At8	Spread of animal abortion causing agents to humans is preventable	SDA/ DA/ N/ A/ SA
At9	Animal health care providers can handle abortion outbreaks very well	SDA/ DA/ N/ A/ SA

Practice subscale items		
P1	Assist animal delivery with protected hands	yes = success / no = failure
P2	Wash hands with soap after assisting animal delivery	yes = success / no = failure
P3	Avoid any contact with aborted material	yes = success / no = failure
P4	Collect aborted fetus and placenta with protective wear	yes = success / no = failure
P5	Always cover hands while touching animal birth products	yes = success / no = failure
P6	Dispose aborted fetus and placenta properly (bury or burn)	yes = success / no = failure
P7	Remove retained placenta manually	yes = failure / no = success
P8	Assist new-born with protected hands	yes = success / no = failure
P9	Wash hands with soap after assisting new-borns	yes = success / no = failure
P10	Suck new-born's noses to remove mucus	yes = failure / no = success
P11	Remove manure from barn regularly	yes = success / no = failure
P12	Take different prevention measures to stop animal abortion outbreak	yes = success / no = failure
13	Report abortion outbreak	yes = success / no = failure
P14	Visit veterinary clinic in case of animal abortion	yes = success / no = failure
P15	Cull frequently aborting animals	yes = success / no = failure

SDA = strongly disagree; DA = disagree; N = neutral; A = Agree; SD = strongly.

The interviews were conducted by four trained veterinarians and animal production experts from the National Agricultural Research System (NARS) who spoke the local language of the respective study sites. They received training on the survey instrument, interview approach, and digital recording of responses. The training ensured a common understanding of the meaning of each question and in what way to ask participants.

### 2.4 Ethical approval and consent from participants

All the procedures for this study were conducted in accordance with a protocol approved by Addis Ababa University, College of Veterinary Medicine and Agriculture (VM/ERC/05/08/11/2018). The farmers/pastoralists were informed about the purpose of the study and the approximate time the interview will take, and their oral informed consent was sought before their participation in the survey.

### 2.5 Data analysis

Descriptive statistics were used to summarise the demographic characteristics and item scores. The item mean scores were transformed to a 0–100 scale. Responses for attitude items were dichotomized into “undesirable” attitudes by combining “strongly disagree” and “disagree” responses and a “desirable” attitude by combining “strongly agree” and “agree” responses. “Neither disagree nor agree” responses were removed during the analysis. A mixed-effect linear regression model was fitted to predict the effect of participants' demographic characteristics on mean scores of knowledge, attitudes, and practices subscales using kebele as a random effect.

The internal consistency of the subscales was assessed by Cronbach's α coefficient, where a Cronbach's α ≥0.7 was considered as acceptable. A value of Cronbach's α > 0.8 was an indicator of good reliability and Cronbach's α between 0.7 and 0.8 indicated adequate reliability. Subscales with Cronbach's α values below 0.5 indicated unacceptable internal consistency [11,55].

Unidimensionality of the three subscales was assessed using the sign of biserial correlations coefficient and confirmatory factor analysis. Negative point-biserial correlations identified potentially problematic items that were subsequently excluded from further analysis. Furthermore, the size of eigenvalues, scree plots, and the magnitude of items loading from the first factor from the factor analysis were used to evaluate the unidimensionality [44,63].

Pearson's correlation coefficient was used to measure the relationship between subscales [53]. The absolute value indicated the strength of the relationship and the sign indicated a positive or negative relationship. Coefficient values between 0.8 and 1.0 indicated a very strong relationship, 0.6 to 0.8 indicated a strong relationship, 0.4 to 0.6 a moderate relationship, 0.2 to 0.4 weak relationship and a value between 0 and 0.2 indicated very weak to no relationship.

A unidimensional two-parameter logistic (2-PL) model was used to evaluate the probability of a person appropriately respond an item with a provided zoonotic disease knowledge, prevention practices level and attitudes towards the risks. This model is represented by the following equation [16]:

$$\mathrm{Pij}\;\left(\mathrm{ui}=1|\theta =t\right)=1/1+\exp\left[-1.7\mathrm{ai}\left(t-\mathrm{bi}\right)\right]$$

where, a_i_ is the discrimination parameter for item i (*i* = 1, …, n), b_i_ is the difficulty parameter for item i, u_i_ is the response of the person with trait level θ (theta) to item i, and 1.7 is a scaling constant.

The item discrimination parameter (a_i_) helps to determine whether the items appropriately distinguished farmers/pastoralists at different levels of the of zoonotic diseases, knowledge, prevention practices, and attitudes. Items with a_i_ values below or equal to 0.7 were removed from subsequent analysis due to low discrimination power.

Item characteristic curves (ICCs) were used to visualize and determine whether an item should be retained or removed. If the ICC of an item was too flat, it was removed because of low discriminatory power between KAPs levels. The item location parameter b_i_ determined the 50% probability of responding to a given item correctly provided the respondent's level of the latent variable (theta θ). The unidimensional latent trait (latent variable) θ was used to assess the ability of a respondent. Predicted values of θ were calculated for every respondent based on their cumulative responses to the KAP questions. The transformed scale of θ had a mean of 0 and a standard deviation of 1 with an arbitrary range to cover the range of zoonotic diseases risk KAP from livestock birth products. The estimation of the parameters was repeated for each subscale after removing unfit items (items with inadequate discrimination).

A test information curve that graphically depicted the amount of information provided the sum of the item information functions at each value of theta for all items in each subscale were plotted.

Differential item functioning (DIF) analysis was performed on the retained items for each subscale to examine whether the items were answered in the same way across respondent groups. The five important subgroupings were: gender (female vs male); literacy level (never went to school vs went to school); primary activity (farmer vs pastoralist); age (less or equal to 35 years vs above 35 years) and intervention status (never seen CRP Livestock animal health intervention vs received CRP Livestock interventions).

Logistic regression was used to determine non-uniform DIF, that is, whether an item favours one group over the other for all values of the latent trait or for only some values of the latent trait. Mantel-Haenszel Tests (MH), which calculated MH X^2^ and odds ratio for dichotomously scored items, were used to test the presence of uniform DIF among the respondent groups. It examined whether an item responded in a better way by one respondent group relative to the other for all values of the latent trait. The statistical significance of the non-uniform DIF of the items was identified by the interaction term. A *p*-value ≤0.05 was used in all analyses to determine the statistical significance. Data analyses were carried out using STATA software program version 15(Texas, USA).

## 3 Results

### 3.1 Characteristics of participants

A total of 323 adult respondents provided complete information during the interview. Table 2 presents the demographic characteristics of the participants. The survey included 80 women (24.77%) and 243 men (75.23%). The mean age of the respondents was 39.5 years (±13.7), with a range from 18 to 85 years. About 60.37% and 39.63% of the participants were farmers or pastoralists, respectively. Agew (39.94%), Borena (39.94%), and Kenbata (20.43%) ethnic groups participated in the study. About 89.78% of the participants were married and 68.42% did not receive any formal education at school. The majority of the respondents (71.52%) reported animal abortion in their herd for the previous two years and 52.94% of respondents had received CRP Livestock animal health interventions.

**Table 2:** Socio-demographic characteristics of participants.

Demography	Category	N (%)	Mean (SD)
Gender	Female	80 (24.77)	
	Male	243 (75.23)	
Age (year)			39.52 (13.68)
Livestock keeping type	MCL farmer	195 (60.37)	
	Pastoralist	129 (39.94)	
Ethnic group	Agew	128 (39.63)	
	Borana	129 (39.94)	
	Kenbata	66 (20.43)	
Literacy level	Never went to school	221 (68.42)	
	Went to school	102 (31.58)	
Marital status	Single	17 (5.26)	
	Married	290 (89.78)	
	Divorced/widowed	16 (4.95)	
CRP Livestock interventions	Not received	152 (47.06)	
	Received	171 (52.94)	
Herd abortion history^a^	No	92 (28.48)	
	Yes	231 (71.52)	

MCL = mixed-crop livestock.

a Abortion history of the herd the last two years.

### 3.2 KAP mean scores

The KAP survey included 48 items representing the three subscales knowledge, attitude, and practice to zoonotic disease risks from livestock birth products among smallholder communities in Ethiopia. The items scores were transformed to a 0–100 scale, and mean item scores for correct answers ranged from 0.31% (±5.6) to 65.42% (±47.6) for knowledge, 12% (±32.5) to 66.4% (±47.3) for attitude, and 4.3% (±20.39) to 90.4% (±29.5) for practice subscales (annexed as supplementary table). Generally, the attitude subscale had the highest total mean score (37.3 ± 28.92%) and the knowledge subscale had the lowest scores (22.4 ± 33.6%) among the three sub-scales (Table 3). However, all subscales had a total mean score below 50%, which could be achieved by chance alone or indicating misperceptions of zoonotic risks. Table 3 summarizes the total mean score of each subscale aggregated by socio-demographic characteristics. Univariable mixed-effect linear regression analysis showed that respondents from Amhara region (Agew community) had a higher total mean score than respondents from Oromia Region (Borena community) (Coef = −15.65, *P* = 0.00) and SNNP region (Kenbata community) (Coef. = − 6.12, *P* = 0.002) for knowledge subscale. However, respondents from SNNP presented a higher desired attitude total mean score compared to respondents in Oromia (Coef = −17.83, *P* = 0.00) and Amhara (Coef. = − 6.10, *P* = 0.153). The practice total mean score was significantly higher for the SNNP respondents than in Oromia (Coef = −11.06, P = 0.00) and Amhara (Coef = −11.84, P = 0.00).

**Table 3:** Knowledge, attitude and practices regarding zoonotic risk compared with socio-demographic variables among communities in Ethiopia.

Demography	Category	N	Knowledge			Attitude			Practice		
			mean	Coef.	p-value	mean	Coef.	p-value	mean	Coef.	p-value
	Overall	323	22.42			37.3			36.20		
Ethnic group	Agew	128	29.92			40.74			38.20		
	Borana	129	14.28	−15.65	0.00	29.01	−12.3	0.001	27.14	−11.0	0.00
	Kenbata	66	23.80	−6.12	0.040	46.84	6.10	0.149	50.04	11.84	0.00
Gender	female	80	22.09			38.63			38.14		
	male	243	22.53	−0.30	0.854	36.86	−2.47	0.506	35.57	−2.58	0.091
Age	≤35	155	21.13			34.49			36.03		
	>35	168	23.62	0.68	0.627	39.89	3.83	0.233	36.36	−0.08	0.925
Literacy level	never went to school	221	21.78			36.20			33.73		
	went school	102	23.82	1.35	0.449	39.68	0.35	0.925	41.57	−1.83	0.126
CRP livestock Intervention	control	152	21.14			35.99			36.18		
	Intervention	171	23.56	0.93	0.595	38.46	1.56	0.649	36.23	−0.84	0.480

### 3.3 Internal consistency

Cronbach's α was calculated for each subscale after removing all items with negative biserial coefficient (5 items). The knowledge and attitude subscales had good internal consistency reliability with Cronbach's α of 0.83 and 0.81, respectively. The practice subscale had a Cronbach's α of 0.51 and thus lower than the minimum acceptable value of 0.7, indicating that this subscale showed inadequate internal consistency reliability.

### 3.4 Correlation between knowledge, attitude, and practice

Through correlation analysis, the Pearson's correlation coefficient (*r*) indicated that there was a moderate positive association between responding correctly in the knowledge section and having the desired attitude (r2 = 0.44, *p* < 0.0001). There was a positive but weak relationship between correctly responding in the knowledge section and self-reported good practice (r^2^ = 0.307, p < 0.0001). Good practices were also positively associated with the desired attitude (r^2^ = 0.18, p < 0.0001).

### 3.5 Item parameter estimates

Factor analysis showed that all subscales were sufficiently unidimensional for the application of unidimensional IRT analysis. A 2PL IRT model was fitted to the data with the marginal maximum likelihood method to estimate the probability of correctly answering an item as a function of the person's ability parameters. The item characteristic curve (ICC) for each item was checked to determine whether an item should be retained or removed. If an item presented a very flat 2pl ICC between −4 to 4, it was removed due to low discrimination power between KAP levels. Accordingly, 6 items were removed from further analysis. The model was then refitted with the remaining items. The items retained for each subscale can be considered as an evaluation scale that measures the zoonotic diseases knowledge, attitude, and prevention practices level of the individual respondent. Parameter estimates of retained items for each subscale are presented in Table 4. Parameter estimates was obtained for 37 items with adequate discrimination power. The mean discrimination parameters for knowledge, attitude, and practice subscales were 2.35 (SD = 1.90), 4.94 (SD = 4.86), and 1.06 (SD = 0.36), respectively. Items in each subscale have a wide range of difficulties and discrimination power. The item with the highest discrimination power (a_i_ = 6.81) in the knowledge subscale was the item *‘animal diseases can be transmitted to you and your family through different routes’*. The item ‘*many of the agents that cause abortion in animals have the potential to cause some in people’* in the attitude subscale had perfect discrimination power (a_i_ = 14.76)_._ In the practice section, the item *‘do you visit the veterinary clinics in case of abortion’* had the highest discrimination parameter (a_i_ = 1·65).

**Table 4:** Discrimination and difficulty parameter from the Item Response Theory analysis of the KAP Scale.

Items	Items contents	Discrimination	Difficulty
Knowledge subscale			
K1	When animals are sick in your flock, you can get the same sickness.	1.17	−0.23
K2	Many animal diseases can be transmitted from animals to humans	4.07	−0.17
K3	Identified the name of three diseases correctly that transmitted from animals to humans	1.19	4.47
K4	Identified the name of only two diseases correctly that transmitted from animals to humans	1.76	1.56
K5	Identified the name of only one disease correctly that transmitted from animals to humans	4.64	−0.03
K6	Please list at least one symptom for any one zoonotic disease in animals	2.32	0.71
K7	Animal disease can be transmitted via different routes	6.82	0.14
K8	Eating uncooked meat can transmit diseases from animals to you	4.37	0.15
K9	Drinking of raw milk can transmit diseases from animals to you	1.37	1.47
K10	Close contact with sick/dead animal can transmit diseases to you	1.77	0.81
K12	Insect bites can transmit animal diseases to you	0.96	4.58
K13	Animal bites can transmit diseases to you	1.41	2.30
K16	Infectious diseases that cause abortion in animals can also cause abortion in humans	1.35	2.23
K17	Abortion causing agents can pass to you through different routes.	1.03	1.89
K18	Identify at least one abortion causing agents correctly that is transmitted from animals to humans	5.82	2.79
K21	Any contact with aborted materials can expose you to diseases	0.87	3.14
K22	Collecting aborted fetues and placenta with bare hand exposes you to diseases	0.73	4.34
K23	Disposing aborted fetuses into the environment can spread diseases	0.79	2.53
	Knowledge subscale average	2.35	1.81

Attitude subscale			
At1	Some animal diseases are dangerous	0.95	−0.78
At2	Diseases that cause animal abortion are serious and need highest consideration	0.82	−0.93
At3	Assisting the animal in delivery time with bare hand can expose you to disease risks.	5.23	1.07
At4	Collecting the aborted fetuses and placenta with bare hands can expose you to disease risks	5.76	1.08
At5	Throwing aborted fetuses and placenta to the environment contribute the spread of the diseases in your farm	10.43	1.01
At6	You are at risk of acquiring diseases from abortion causing agents	2.62	0.99
At7	Many of the agents that cause abortion in animals have the potential to cause some diseases in people.	14.77	1.13
At8	Spread of animal abortion causing agents to humans is preventable	3.62	0.31
At9	Animal health care providers can handle abortion outbreaks very well	0.90	−0.39
	Attitude subscale average	5.01	0.39

Practice subscale			
P1	Assist animal delivery with protected hands	0.76	3.29
P2	Wash hands with soap after assisting animal delivery	0.71	−2.77
P3	Avoid any contact with aborted material	0.76	3.52
P4	Collect aborted fetus and placenta with protective wear	1.62	2.46
P5	Always cover hands cut while touching animal birth products	1.32	2.63
P6	Dispose aborted fetus and placenta properly (bury or burn)	0.89	2.43
P9	Wash hands with soap after assisting new-born	0.77	−1.07
P13	Report abortion outbreak	1.20	0.38
P14	Visit veterinary clinic in case of animal abortion	1.66	1.36
P15	Cull frequently aborting animals	0.93	−0.75
	Practice score average	1.06	1.15

Mean difficulty parameters for subscales ranged from 0.38 (0 0.862) to 1.82 (SD = 1.57). The attitude section had the easiest items while the knowledge questions were harder. The mean of the practice section location parameters was 1.14 (SD = 2.12). Difficulties expressed through the location parameters ranged from −0.231 to 4.58 for knowledge items; −0.93 to 1.13 for attitude items; −2.76 to 3.52 for practice items.

Among the knowledge items, the easiest was *‘when animals are sick in your flock, you might get the same sickness*’ and the most difficult was ‘*insect bite can transmit animal diseases to you’*. The attitude item with the lowest location (difficulty) parameter was *‘diseases that cause animal abortion are serious and need the highest consideration’*, and the item with the highest location parameter power was item ‘*many of the agents that cause abortion in livestock have the potential to cause some disease in people’*. *‘Washing hands with soap after assisting animal delivery’* was the easiest practice item, whereas *‘avoiding any contact with aborted materials’* was the hardest, respectively the least common practice for the farmers/pastoralists to implement.

**Fig. 2:**
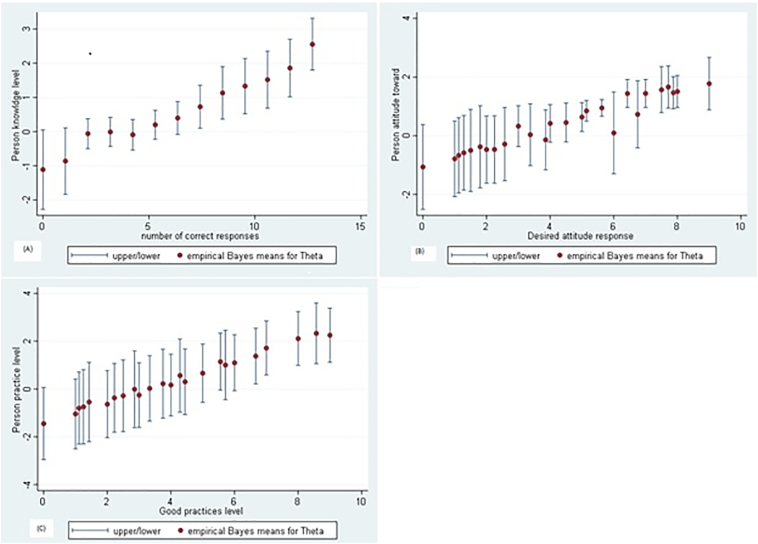
Plot of predicting subject scores (thetas) vs. proportion of correct response of knowledge (A), attitude (B) and practice (C) subscales.

Fig. 2 presents the predicted θ scores for each respondent compared with the probability of correct responses in the respective KAP subscale. There was a difference in zoonotic diseases knowledge, attitude, and risk prevention practice level among respondents. The probability of someone answering all correct ranged between knowledge of the level of −1.1 to 2.6, attitude level of −1.2 to 1.8, and practice level of −1.45 to 2.56 (Fig. 2).

**Fig. 3:**
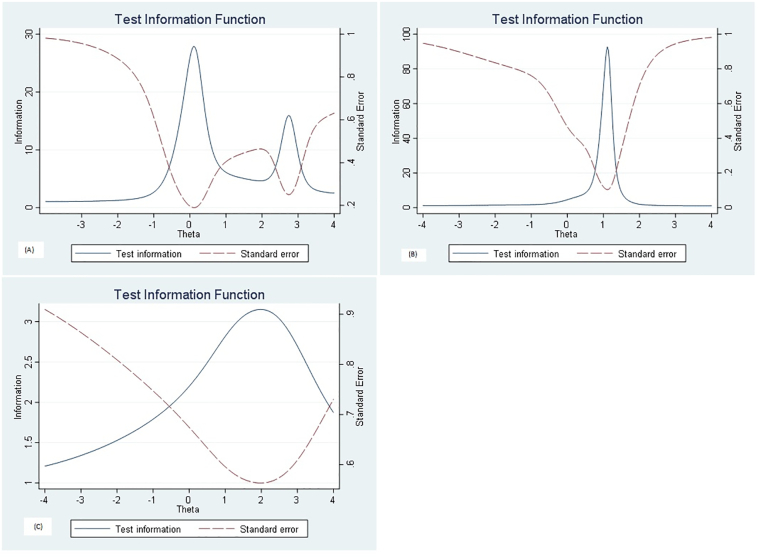
Test information for knowledge (A), Attitude (B) and practice (C) subscales.

Fig. 3 (A-C) presents the test information functions (solid lines) and standard errors (broken lines) to the knowledge(a), attitude (b), and practice(c) subscales. The KAP tool provided maximum information for respondents with θ between −0.8 to 0.8 for knowledge, 0.7 to 1.5 for attitude and − 0.7 to 2 for the practice subscale, respectively.

### 3.6 Differential item functioning

We examined differential item functioning (DIF) in concerning to gender, literacy, primary livelihood activity, age and recent exposure to animal health related interventions.

Table 5 presents items that had non-uniform DIF for each of the covariates based on a 5% significance level for KAP subscales. The results of the logistic regression analysis showed that one item had DIF associated to gender, six items related to age, one item associated with literacy level, three items associated with primary activity, and five items associated with intervention status for the knowledge section. For attitude, two items had DIF linked to gender, age, and primary activity, one item had DIF linked to intervention status, and none of the items had DIF related literacy level. For the practice subscale, one of the items had DIF linked to gender and primary activity, two items had DIF related to age and intervention status, and none of the items presented DIF related to literacy level.

**Table 5:** Logistic regression DIF analysis findings for zoonotic diseases KAPs subscales.

Item⁎	Gender		Age		Literacy level		Activities		Intervention status	
	X^2^	P-value	X^2^	P-value	X^2^	P-value	X^2^	P-value	X^2^	P-value
Knowledge subscale										
K1	2.52	0.1127	0	0.9939	0.9	0.3428	22.5	0.00	0.13	0.7136
K2	0.18	0.6755	4.81	0.0283	1.24	0.265	0.42	0.5188	1.14	0.2846
K7	0	0.9972	8.8	0.003	9.71	0.0018	0.33	0.5642	0	0.9814
K9	2.51	0.1131	4.26	0.0391	1.45	0.2288	4.81	0.0283	3.99	0.0457
K10	1.36	0.2439	0.44	0.5067	0.01	0.9026	17.02	0.00	4.21	0.0403
K13	0.69	0.4055	5.39	0.0203	2.56	0.1099	0.03	0.8602	9.45	0.0021
K17	4.29	0.0384	0.28	0.5972	1.44	0.2304	0.32	0.5744	0.01	0.932
K21	0.19	0.6603	0.59	0.4419	2.18	0.1394	1.64	0.201	17.47	0.00
K22	0	0.9597	4.9	0.0269	0.07	0.7938	0.85	0.3569	0.3	0.5808
K24	2.21	0.1369	3.93	0.0474	0.02	0.8914	2.46	0.117	7.59	0.0059

Attitude subscale										
At2	6.72	0.0096	0.26	0.6088	2.63	0.105	2.77	0.0961	2.04	0.1528
At3	.	.	0.82	0.3658	2.51	0.1133	5.36	0.0206	0	0.9927
At6	0.04	0.8487	7.12	0.0076	0.29	0.5927	1.12	0.2892	5.65	0.0175
At8	7.48	0.0062	4.15	0.0415	0.01	0.9097	4.97	0.0258	3.6	0.0577

Practice subscale										
P6	3.15	0.0758	4.54	0.033	0.53	0.4648	.	.	0.63	0.4284
P9	0.04	0.8409	0.01	0.9147	3.32	0.0686	7.46	0.0063	9.31	0.0023
P13	0.75	0.3857	12.31	0.0005	0.04	0.8477	1.97	0.1599	0.17	0.6776
P14	8.59	0.0034	1.03	0.3102	2.11	0.1468	–	–	1.38	0.2406
P15	2.2	0.1382	1.83	0.1763	0.01	0.9089	0.15	0.694	4.08	0.0433

⁎ items that had DIF for at least one covariate based on a 5% significance level were presented.

Table 6 presents the items that had a uniform DIF for each of the covariates based on a 5% significance level. The Mantel-Haenszel DIF test for knowledge items showed that the item ‘*when animals are sick in your flock, you might get the same sicknesses*’ answered in a better way by respondents groups who never received formal education (odds ratio = 2.7), pastoralists (odds ratio = 2.1879) and respondents who have not involved in any intervention form CRP Livestock (odd ratio = 3.57) compared to their counterparts. Crop-livestock farmers had 3.44 higher odds to identify at least one zoonotic disease correctly (item K5) and 2.87 and 2.56 higher odds to answer items related to animal disease transmission routes (K7) and eating uncooked meat can transmit diseases from animals (K8) correctly compared to pastoralists.

**Table 6:** Mantel-Haenszel DIF analysis findings for zoonotic diseases KAPs scales.

	Gender		Age		Literacy level		Activity		Intervention	
Item⁎	OR	P-value	OR	P-value	OR	P-value	OR	P-value	OR	P-value
Knowledge subscale										
k1	0.83	0.7126	1.07	0.917	0.3735	0.0042	2.1879	0.0074	0.2803	0.000
k5	1.08	0.9236	0.82	0.7877	1.6914	0.3102	0.2975	0.0121	1.7457	0.2763
k7	0.94	0.8513	1.01	0.8282	0.6157	0.3625	0.348	0.0432	0.4814	0.2182
k8	1.12	0.9896	0.74	0.6061	0.7941	0.7564	0.3974	0.0459	1.2099	0.8042
k9	0.34	0.0121	0.42	0.0211	3.1236	0.0007	1.9411	0.1962	1.6575	0.2356
k10	0.95	0.9585	0.49	0.0428	1.6502	0.1701	2.5655	0.0632	0.9151	0.9179
k13	0.49	0.3424	0.22	0.0531	1.2365	0.9394	2.2198	0.0468	0.7863	0.8443
k17	1.50	0.4047	1.63	0.2567	0.3362	0.013	0.3624	0.0898	0.7835	0.5956

Attitude subscale										
At9	0.17	0.007	2.63	0.0583	0.9377	0.9137	0.99	0.8330	0.8695	0.9129

Practice subscale										
P9	0.36	0.0467	0.92	0.9464	0.5323	0.1606	2.0395	0.4775	1.1641	0.7594
P11	1.78	0.3963	0.34	0.0515	0.4043	0.2161	–	–	3.0654	0.0489
P13	1.74	0.2359	2.35	0.0184	1.8664	0.1981	0.0076	0.000	0.7085	0.3832

⁎ items that had DIF for at least one covariate based on a 5% significance level were presented.

Female respondents, respondents with formal education, and respondents aged 35 or below had 2.94, 2.38, 3.12 times higher odds to respond correctly to the item ‘*drinking of raw milk can transmit diseases from animals to you*’ compared to their counterparts. Being aged 35 years or less also increased the odds of responding correctly to the item *‘close contact with the sick/dead animal can transmit disease to you’* by 2.04 times. Respondents who engaged in mixed crop-livestock farming had 2.21 higher odds to respond to the item *‘animal bite can transmit diseases to you’*) correctly compared to pastoralists. Finally, respondents who never went to school had 3.05 times higher odds to correctly respond to the item *‘abortion causing agents can pass to you through different routes’* compared to those with formal education.

The Mantel-Haenszel DIF test for attitude items showed that only one item had a uniform DIF related to gender (Table 6). Female respondents had 5.88 times higher odds to have a positive attitude for the item *‘animal health care providers can handle abortion outbreaks very well’*.

The results of the Mantel-Haenszel DIF test on practice items showed that women had 2.78 times higher odds to wash hands with soap after assisting new-borns than men. Respondents aged 35 years or less and those who had been involved in CRP Livestock health intervention had 2.94 and 3.06 higher odds to remove manure from the barn regularly. Respondents who were older than 35 years age and who were involved in mixed-crop livestock farming had 2.35- and 131.57-times higher odds to report an abortion outbreak than their counterparts, respectively.

## 4 Discussion

To the best of our knowledge, this is the first study attempting to describe the knowledge, attitude, and prevention practices of zoonotic disease risk from livestock birth products in Ethiopia. The current study revealed overall low zoonotic disease knowledge, low attitude towards zoonotic disease risk, and common risk behaviours among smallholder farmers and pastoralists. Even though the majority of the respondents reported sheep and goats' abortion during the two years before the interview, the causes of abortion, their transmission modes, preventive actions, and their public health significance are rarely known and understood by farmers and pastoralists. Previous studies in Ethiopia [36,64] and elsewhere [43,65] have also described knowledge gap on the public health risk of zoonotic abortion-causing agents such as Brucella, Leptospira, Toxoplasma, Chlamydophila and Coxiella. This low level of awareness of zoonotic diseases among communities may be the consequence of low information and awareness about the burden and transmission of the disease among veterinary and public health professionals, inaccessibility of public health centres, and lack of trained manpower in health education [21,43,51,59]. In developing countries such as Ethiopia, the lack of appropriate diagnostic tools for the detection and isolation of pathogenic agents limits reliable qualitative and quantitative information on the burden of zoonotic disease [48]. This lead to underestimation of the burden and the impact of zoonotic diseases on the community among policy makers and donors, which in turn is an obstacle to develop and implement appropriate policies to assess and manage zoonotic disease risk when there are other competing public health priorities [10]. Therefore, generating reliable information on zoonotic disease burden needs to be given higher priority to increases the awareness level of government agents, funders, and other stake holders [59]. Increased awareness will help to promote cost-effective integrated approaches to address these knowledge gaps to reduce the risk of zoonotic infection to livestock producers and livestock products consumers along value chain within existing health and agricultural systems.

It is generally assumed that expanding community-based livestock health education and promotion activities has a key role in improving the awareness of livestock framers towards zoonotic disease risk. Consequently, the behavioural practices of the farmers improved and the likelihood of human exposure from livestock significantly decreased [26,47,49,60]. The Federal Ministry of Health of Ethiopia launched the Health Extension Program in 2003 to increase the knowledge and skills of communities and households to deal with preventable diseases and to promote health in rural villages of Ethiopia [7]. While this program brings in significant difference on prevention of transmissible diseases, family planning and the hygiene and sanitation of the environment [2,7,9,52], there remain important gaps in zoonotic disease transmission prevention. Integrating zoonotic diseases control training into this program with clear linkages to livestock health management would allow delivering accurate public health information about the zoonotic disease risk to the local community in rural areas of Ethiopia. Development agents are well known by the local community and their massages are positively taken. Therefore, it is important to consider those agents for conveyance of information related to zoonotic disease transmission routes, prevention methods and animal health management related issues for the farmers [58].

Incorrect perceptions and attitudes towards the prevention of zoonotic disease from animal birth products, such as assisting and dealing with animal abortion with bare hands and improper disposal of aborted fetus and placenta, strongly support the need for culturally appropriate health education in rural communities. Therefore, it is important to change the attitude of the community to improve their behavioural practice towards zoonotic diseases transmission prevention practices [46]. The finding of this study suggested that establishing a desired attitude on impact of those diseases on public health and their mitigation strategies among the community is vital to reduce the transmission of zoonotic agents from animals to humans.

The majority of farmers and pastoralists did not implement appropriate risk prevention practices. Comparable findings were also obtained from Pakistan [5], Tajikistan [39], and Egypt [29]. Poor knowledge of animal owners could be one explanation for these high-risk behavioural practices. Moreover, farmers and pastoralists do not use personal protective equipment when dealing with animal abortion due to the limited availability that equipment in their areas.

The mixed model regression analysis indicated an important difference in zoonotic disease knowledge, attitude, and practices across regions. This might be related to the difference in availability and accessibility of public and animal health facilities in studied agroecology. Mixed crop-livestock farmers tend to have better understanding of the problems since they have relatively a better access to information because of better infrastructure such as health centers and roads. Borana pastoral communities and their livestock have a high level of mobility which hampers access to resources, health services and information due to limited infrastructures in the area, their living style and societal restrictions [51]. In contrast, crop-livestock mixed farmers have a better access to veterinary services such as vaccination and health education through different campaigns implemented by government and non-government agencies [30].

In the present study, the IRT method was used to develop scales to evaluate zoonotic KAP items. IRT models characterize items with different difficulty levels and measure discriminatory power based on responses and identify items that are not appropriate to be included in the scale for evaluation [35]. In this study, all three subscales met the assumption that the underlying trait measured was unidimensional and all, except the practice subscale, showed good reliability with acceptable Cronbach's α values. Moreover, the knowledge and attitude subscales were positively correlated with the practice subscale. Knowledge and risk perceptions of zoonotic causes of abortion among high-risk community groups are crucial in influencing behavioural practices in preventing its transmission form animals to human. Effective public education strategy for zoonotic diseases demonstrated a detectable positive effect on risk perception and possible actions that can be taken to safeguard human health through appropriate hygienic measures and preventive practices [14,27,31,58].

Our results found moderate to very high discrimination mean values of KAP subscales, which shows strong consistency between the items and the KAP levels of the respondents [8,24]. The difficulty parameter estimates showed that the attitude section had the easiest items, and the knowledge section was comparably more difficult. This result indicated that it can be possible to have a positive attitude towards zoonotic diseases risks with some level of knowledge on zoonotic disease from livestock birth products. Items in the knowledge subscale required a higher level of knowledge to be answered correctly. This indicated health education on prevention and control of zoonotic diseases was not provided for the animal owners or correct levels of knowledge on zoonotic diseases at livestock birth were not attained. These results provide vital information for future interventions and illustrate a critical need to improve farmers' and pastoralists' knowledge.

Predicted subject scores (θ) indicated that the probability of correct responses increased consistently with individual knowledge, attitude, and practice level. However, the attitude subscale needs additional questions to be able to better discriminate between attitude levels. The test information curve showed that the knowledge subscale questions provided more precise information within the medium knowledge scores. However, attitude and practice questions presented more precise information within the higher trait levels of farmers and pastoralists. The KAP scales developed in this study can thus be used in future studies, such as, for example, for the evaluation of interventions aimed at improving public awareness on zoonotic diseases from livestock birth and abortion.

The DIF analysis found that 19 of 37 (51.35%) and 12 of 37 (32.43%) items of the final version of the KAP survey differed depending on subgrouping of the respondents. The presence of DIF in the majority of KAP questions in this study might be attributed to the lack of equal understanding of zoonotic disease risks and their perception about the risks and risk prevention practices among the respondents. It appears that there are discrepancies between the real risks associated with animal abortion and their perception by the public. This difference could be due to communication inequalities at the grassroot level.

Moreover, some respondent groups had a higher awareness regarding zoonotic diseases from livestock birth products, which might be attributed to their desire to obtain health information from media and professionals. Farmer and pastoralist perceptions about zoonotic disease risk from livestock is guided by socio-environmental barriers, beliefs and may often be misinformed and incorrect [[3], [4], [5],^15^].

The role and location of the households might determine livestock owners' awareness, disease identification skills, and preventive behavioural practices, which need attention during community health education program development. For example, women and men have different experiences and capacities in animal management and husbandry. Women have been predominantly involved in the milking and processing of milk as well as the care of sick animals and aborted animals [32,45,62]. Similarly, women are responsible for milking in Borana pastoral communities [4]. Therefore, women involved in milking and milk processing daily have a better chance to observe foreign materials such as dungs or pus from animals with mastitis. This increases the concern of women towards milk hygiene and encourages women to boil milk before serving, which would help prevent most milk-borne diseases. This implies the need for community health education programs which target specific groups such as youth, women, farmers, pastoralists, or community leaders. This in turn requires to build the capacity of development agents, medical and veterinary doctors, and technical personals and calls for more efficient public health education at the grassroots level [26,58]. Appropriately prepared targeted public information materials such as pamphlets and posters can be used to communicate information on diseases to local communities and to encourage them to adopt better risk management practices [27]. More promising to achieve lasting practice change, however, would be discussions with communities to clarify myths and find context-specific solutions that are accepted by the communities [38,41]. Moreover, the utilization of local mass media such as a radio to disseminate public information also plays a key role in continued efforts to prevent and control zoonotic diseases and promoting zoonotic control interventions [1,59,65].

### 4.1 Limitation of study

Even though interviewers received training to ensure a common understanding of the meaning of each question and in what way to ask them, the questions may have been interpreted incorrectly by the respondents. The validity of the questions was ensured through expert consultation and pre-testing with a pilot group of farmers before the survey. Attempts were also made to make sure that the farmers and pastoralists understood all items correctly before they responded. The items included were based on expert knowledge and literature review but did not include in-depth qualitative research with communities at first. Adding this step may help to identify additional items to be added to the KAP tool in the future.

## 5 Conclusion

The present study evaluated the knowledge, attitudes, and prevention practices of zoonotic disease from livestock birth products in rural Ethiopia. The KAP tool developed showed high validity and reliability. This study highlighted substantial knowledge gaps and high-risk behavioural practices towards zoonotic disease risk from livestock births products, which are an important contributing factor for zoonotic disease infection. Differential item functioning test showed that more than half of the items in the final version of the KAP scale had DIF related to at least one covariate, which indicated all items were not equally addressed by the respondents. The low mean scores recorded for all subscales and the presence of DIF in the majority of the items highlight the need for targeted community health education programs to minimize the transmission of zoonotic pathogens from livestock birth products.

## Authors' contributions

**Gezahegn Alemayehu**: Conceptualization, Methodology, Formal analysis, Investigation, Data curation, Writing - original draft. **Barbara Weiland**: Funding acquisition, Validation, Writing - reviewing and editing, Supervision. **Gezahegne Mamo**: Supervision, Writing - reviewing and editing. **Hiwot Desta**: Data curation, Validation. **Biruk Alemu**: Data curation, Validation, Writing - reviewing.

## Declaration of Competing Interest

The authors declare that they have no competing interests.

## Appendix A Supplementary data

Supplementary material Image 1
