# Supplementary material for: Knowledge, attitude, and practices to zoonotic disease risks from livestock birth products among smallholder communities in Ethiopia
Source: One Health. 2021 Jan 30;12:100223. doi: 10.1016/j.onehlt.2021.100223 (PMC7879039; doi:10.1016/j.onehlt.2021.100223)
Supplement: Supplementary file 1 — Supplementary material [file mmc1.docx]

Supplementary table1. Mean items scores of KAPs survey on zoonotic disease risks from livestock births

| **Item no** | **Item content** | **Mean (%)** | **SD (%)** |
| --- | --- | --- | --- |
| **Knowledge subscale items** | | | |
| K1 | When animals are sick in your flock, you can get the same sickness. | 55.4 | 49.8 |
| K2 | Many animal diseases can be transmitted from animals to humans | 57.0 | 49.6 |
| K3 | Identified the name of three diseases correctly that transmitted from animals to humans | 52.0 | 50.0 |
| K4 | Identified the name of only two diseases correctly that transmitted from animals to humans | 13.3 | 34.0 |
| K5 | Identified the name of only one disease correctly that transmitted from animals to humans | 0.9 | 9.6 |
| K6 | Please list at least one symptom for any one zoonotic disease in animals | 28.8 | 45.3 |
| K7 | Animal disease can be transmitted via different routes | 46.1 | 49.9 |
| K8 | Eating uncooked meat can transmit diseases from animals to you | 45.8 | 49.9 |
| K9 | Drinking of raw milk can transmit diseases from animals to you | 18.0 | 38.4 |
| K10 | Close contact with sick/dead animal can transmit diseases to you | 28.2 | 45.1 |
| K11 | You can get infection from environment contaminated from secretions of sick animals | 1.9 | 13.5 |
| K12 | Insect bite can transmit animal diseases to you | 1.9 | 13.5 |
| K13 | Animal bites can transmit diseases to you | 7.4 | 26.3 |
| K14 | Animal abortion can cause a serious economic and public health problem | 65.4 | 47.6 |
| K15 | Abortion in animals can be caused by agents that spread between animals | 39.6 | 49.0 |
| K16 | Infectious diseases that cause abortion in animals might cause abortion in humans | 8.7 | 28.2 |
| K17 | Abortion causing agents can pass to you through different routes | 16.4 | 37.1 |
| K18 | Name at least one abortion causing agents that is transmitted from animals to humans | 0.3 | 5.6 |
| K19 | Assisting animals during parturition with bare hand exposes you to diseases | 7.1 | 25.8 |
| K20 | Assisting new-borns right after delivery exposes you to diseases | 4.3 | 20.4 |
| K21 | Any contact with aborted materials can expose you to diseases | 8.0 | 27.2 |
| K22 | Collecting aborted fetuses and placenta with bare hand exposes you to diseases | 5.0 | 21.7 |
| K23 | Disposing aborted fetuses into the environment can spread the diseases | 13.0 | 33.7 |
| K24 | Animal abortion can be prevented | 14.2 | 35.0 |
|  | **Attitude subscale items** | | |
| At1 | Some animal diseases are dangerous for people | 65.4 | 47.6 |
| At2 | Diseases that cause animal abortion are serious and need highest consideration | 66.4 | 47.3 |
| At3 | Assisting the animal in delivery with bare hand can expose you to disease risks. | 15.6 | 36.3 |
| At4 | Collecting the aborted fetuses and placenta with bare hands can expose you to disease risks | 15.2 | 36.0 |
| At5 | Throwing aborted fetuses and placenta to the environment contribute the spread of the diseases in your farm | 14.9 | 35.7 |
| At6 | You are at risk of acquiring diseases from abortion causing agents | 21.0 | 40.8 |
| At7 | Many of the agents that cause abortion in animals have the potential to cause disease in people. | 12.0 | 32.5 |
| At8 | Spread of animal abortion causing agents to humans is preventable | 42.4 | 49.5 |
| At9 | Animal health care providers can handle abortion outbreaks very well | 58.4 | 49.4 |
| **Practice subscale items** | | | |
| P1 | Assist animal delivery with protected hands | 9.3 | 29.1 |
| P2 | Wash hands with soap after assisting animal delivery | 85.8 | 35.0 |
| P3 | Avoid any contact with aborted material | 8.0 | 27.2 |
| P4 | Collect aborted fetus and placenta with protective wear | 5.0 | 21.7 |
| P5 | Always cover hands while touching animal birth products | 5.9 | 23.6 |
| P6 | Dispose aborted fetus and placenta properly (bury or burn) | 13.0 | 33.7 |
| P7 | Remove retained placenta manually | 18.6 | 39.0 |
| P8 | Assist new-born with protected hands | 67.7 | 46.9 |
| P9 | Wash hands with soap after assisting new-borns | 39.7 | 49.0 |
| P10 | Suck new-born’s noses to remove mucus | 16.3 | 37.0 |
| P11 | Remove manure from barn regularly | 64.0 | 48.1 |
| P12 | Take different prevention measures to stop animal abortion outbreak | 90.4 | 29.5 |
| 13 | Report abortion outbreak | 4.3 | 20.4 |
| P14 | Visit veterinary clinic in case of animal abortion | 67.4 | 46.9 |
| P15 | Cull frequently aborting animals | 53.9 | 49.9 |
